# Carbapenem-resistant Enterobacterales (CRE) colonisation as a predictor for subsequent CRE infection: A retrospective surveillance study

**DOI:** 10.4102/sajid.v40i1.687

**Published:** 2025-01-28

**Authors:** Courtney M. Tubb, Marco Tubb, Jonathan Hooijer, Rispah Chomba, Jeremy Nel

**Affiliations:** 1 Department of Internal Medicine, Faculty of Health Sciences, University of the Witwatersrand, Johannesburg, South Africa; 2 Department of Clinical Microbiology and Infectious Diseases, School of Pathology, University of the Witwatersrand, Johannesburg, South Africa

**Keywords:** CRE Enterobacterales, CRE colonisation, CRE infection, Antibiotic, Carbapenamase, carbapenem resistance

## Abstract

**Background:**

Carbapenem-resistant Enterobacterales (CRE) are associated with significant morbidity and mortality. Carbapenem-resistant Enterobacterales colonisation is an important prerequisite for infection, and its surveillance is crucial to reduce spread. However, data from South Africa are limited.

**Objectives:**

We aimed to determine CRE colonisation prevalence, the incidence of subsequent CRE infections and the risk factors associated with each.

**Method:**

We retrospectively reviewed hospital records from 686 patients admitted to a medical high-care ward at a tertiary hospital in Gauteng, South Africa, between October 2019 and May 2022. Patients were grouped by CRE colonisation status on arrival and discharge. Data on comorbidities, indwelling devices and antibiotic exposure were collected.

**Results:**

The prevalence of CRE colonisation was 12.4% (95% confidence interval [CI]: 10.1–15.1), with *Klebsiella pneumoniae* (81.2%) being the most common CRE isolated and OXA-48-like enzymes (94.5%) being the most frequent carbapenemase detected. Risk factors for CRE colonisation on the univariate analysis included exposure to antibiotics (odds ratio [OR]: 2.21; 95% CI: 0.98–4.96, *P* = 0.048) and presence of a central venous line (OR: 6.33; 95% CI: 1.78–22.46, *P* = 0.001). Of patients colonised with a CRE, 21.2% subsequently developed a culture-positive infection within 180 days from the initial colonisation result and the majority within 30 days. These infections were mostly CREs (OR: 4.0, 95% CI: 1.3–12.7), and where the infections were CREs, the causative CRE organism and carbapenemase subtype were identical in each case.

**Conclusion:**

Our study documented higher CRE prevalence rates than those previously reported from South Africa. Given the association between CRE colonisation and subsequent infection, urgent measures are required to reduce CRE colonisation rates. As the organism and carbapenemase detected in the initial colonisation and subsequent CRE infection were closely related, knowledge of prior CRE colonisation may assist clinicians with antibiotic choice if patients present with an infection within 30 days of CRE colonisation.

**Contribution:**

This study reports higher CRE colonization rates in South Africa than previously documented, highlighting the urgent need to reduce colonization. The close genetic link between CRE colonization and subsequent infection suggests that knowledge of prior colonization can guide clinicians in selecting effective antibiotics, particularly for infections occurring within 30 days. These findings support targeted interventions to address the rising CRE threat.

## Introduction

Antimicrobial resistance (AMR) causes significant morbidity and mortality and is considered one of the leading threats to public health in the 21st century.^1,2,3^ Antimicrobial resistance was linked to 4.95 million deaths globally in 2019, with 1.27 million of these directly attributed to drug-resistant infections.^2,3^ Up to 10 million deaths because of AMR are predicted by 2050. Sub-Saharan Africa has been highlighted as a high-burden region for AMR-associated death, and carbapenem-resistant Enterobacterales (CRE) are a significant contributor to this.^2,3,4^ Carbapenem-resistant Enterobacterales bloodstream infections are associated with high mortality, ranging from 24% to 50%.^5,6,7,8^ The Group for Enteric, Respiratory and Meningeal Diseases Surveillance in South Africa (GERMS-SA) reported a 57% rise in CRE bacteraemia cases nationally between 2018 and 2019. *Klebsiella pneumoniae* was the predominant organism cultured (80%), followed by *Serratia marcescens* (5%), *Enterobacter cloacae* (5%) and *Escherichia coli* (5%).^9^

Carbapenem-resistant Enterobacterales exert their resistance to carbapenems through many mechanisms, with carbapenemase production being the most common.^10,11^ Carbapenemase-producing Enterobacterales (CPEs) are classified according to the Ambler classification and their subtypes differ geographically.^11^ Oxacillinase-48 (OXA-48) and New Delhi metallo-β-lactamase (NDM) are the most predominant CPEs in South Africa.^9^

The CRE colonisation is most frequently identified through a positive rectal swab, and colonisation is thought to be a prerequisite for infection.^12^ Carbapenem-resistant Enterobacterales rectal carrier surveillance and early identification is therefore an important tool to prevent CRE spread.^12^ Identifying the risk factors associated with CRE infection in CRE-colonised patients may help to address modifiable risk factors and direct empiric antibiotic choices, thus reducing the morbidity and mortality associated with these infections.^13,14,15^ A 2016 international meta-analysis study reported an overall 16.5% risk of CRE infection in patients colonised with CRE and a mortality rate of 10% in patients either colonised or infected with CRE.^14^ Diabetes mellitus, ICU admission, use of central venous catheters (CVC) and prior antibiotic exposure (particularly fluoroquinolones and metronidazole) have been identified as risk factors associated with CRE infection.^13,14,15^

Data on CRE colonisation prevalence in South Africa are limited. To date, there have been only two published reports on CRE colonisation prevalence, both based on the data collected over 8 years ago, with reported rates of 0.23% and 5.12%, respectively.^16,17^ Given the rising rates of CRE infections reported in recent years, more up-to-date colonisation data are urgently needed. A recent study indicated that 33% of patients with a CRE infection had either a prior CRE infection or documented CRE colonisation within the preceding 12 months.^18^ However, the incidence of subsequent CRE infections among patients colonised with CRE has yet to be studied in an African setting. We aimed to determine CRE colonisation prevalence in a South African tertiary academic hospital’s high-care unit, the incidence of subsequent CRE infections and the risk factors associated with each.

## Research methods and design

We conducted a retrospective surveillance study at Helen Joseph Hospital, a tertiary academic hospital located in Gauteng, South Africa. It is a 550-bed hospital providing services to a catchment area of approximately 1 million people. The medical high-care unit is a six-bed facility that can accommodate medical patients requiring increased monitoring, including inotropic support but not invasive mechanical ventilation.

During the study period, all patients were screened for CRE rectal colonisation with a rectal swab on admission and discharge from the high-care unit. Helen Joseph’s laboratory is part of the National Health Laboratory Service (NHLS). Rectal swabs were inoculated in a brain heart infusion (BHI) broth with a 10 µg ertapenem disc and incubated (aerobically overnight at 35 ± 2°C) for 16–18 h.^19^ Broths were subcultured onto MacConkey agar plates. Pre-enrichment of screening cultures was used as an additional means to increase the sensitivity of MDR Enterobacterales detection.^20,21,22^ Antibiotic susceptibility testing (AST) was performed on each unique Gram-negative colony using carbapenem discs (ertapenem, meropenem and imipenem). Isolates that tested non-susceptible by Clinical and Laboratory Standards Institute criteria were classified as CRE. Carbapenemases were identified through the RESIST-4 O.K.N.V. (Coris BioConcept, Gembloux, Belgium) assay. The RESIST-4 O.K.N.V. assay can identify oxacillinase 48 (OXA-48), NDM, *K. pneumoniae* carbapenemase (KPC), Verona integron-encoded metallo-beta-lactamase (VIM) and Imipenemase metallo-beta-lactamase (IMP) carbapenemases. Organisms were identified using Vitek^®^2 instrument (bioMérieux Inc., Durham, NC, USA).

For the purpose of this study, patients were defined as CRE colonised if rectal swab results were CRE positive on admission, or discharge or both. All patients aged 14 years and older who were admitted to this medical high-care between October 2019 and May 2022 were retrospectively assessed for inclusion. Patients without a traceable surveillance rectal swab were excluded from the analysis.

Electronic laboratory records for all included CRE-colonised patients were screened and any positive blood, urine or sputum cultures within 180 days from initial detection of colonisation (either on admission or discharge) to the medical high-care were identified. A subsequent infection was defined as a positive culture blood culture, a positive urine culture with >10 000 leucocytes/mm^3^ or a positive sputum culture with a positive Bartlett score ≥ 0. No routine surveillance cultures were included.

Data on demographics, medical comorbidities, presence of medical devices (CVC, urine catheters, nasogastric tubes) and antibiotic exposure during the high-care admission were extracted from hospital medical and electronic records. For patients with a positive CRE rectal swab culture, data on the specific CRE organism and associated resistance genes were also collected. Data were collected on a paper-based data collection tool sheet and transferred to an electronic database.

Data were analysed using STATA version 17. We used univariable and multivariable logistic regression to determine the association between a set of predefined demographic and clinical risk factors and a positive CRE rectal swab on discharge among those who had an initial negative CRE swab on admission. Those with an unchanged CRE swab status or with missing data were excluded from this part of the analysis. For the analysis of subsequent CRE-positive cultures, only univariable logistic regression was conducted, as data sparsity precluded multivariate analysis. Associations were determined by odds ratios with 95% confidence intervals (CIs); *p* < 0.05 was considered to be statistically significant.

### Ethical considerations

Approval for this study was obtained from the Helen Joseph Research Committee (reference no.: GP_202211_008) and the Medical Human Research Ethics Committee at the University of the Witwatersrand (reference no.: M221134).

## Results

Six hundred ninety-one patients were admitted to the medical high-care unit between October 2019 and May 2022. Of these, 686 patients (99.3%) had at least one traceable rectal swab screening result (admission swab and/or discharge swab) and were included in the study cohort. The median age of the participants was 45 (interquartile range [IQR]: 34–59), with an equal distribution of males (*n* = 331; 48.3%) and females (*n* = 355; 51.7%). The majority of patients (78%) were admitted between 2020 and 2021, and patients stayed in the high-care unit for a median of 3 days (IQR: 1–6). During their stay, nearly two-thirds (62.6%) of patients had a CVC and almost 30% of patients had a transurethral catheter. One or more comorbidity was present in 27.6% of patients, of which diabetes was the most common, *n* = 116 (16.9%) (Table 1).

**TABLE 1:** Demographic and clinical details of the study cohort (*N* = 686†).

Characteristic	*n*	%	Median	IQR
Age in years (*N* = 683)	-	-	45	34–59
Male	331	48.3	-	-
**Year of admission**	-	-	-	-
2019	57	8.4	-	-
2020	288	42.2	-	-
2021	246	36.0	-	-
2022	92	13.5	-	-
Duration of admission in days (*N* = 682)	-	-	3	1–6
**Devices used during admission**	-	-	-	-
Central venous catheter (*N* = 521)	326	62.6	-	-
Transurethral catheter	201	29.3	-	-
Nasogastric tube	34	5.0	-	-
Surgical drain	2	0.3	-	-
No devices	7	1.0	-	-
**One or more underlying comorbidities**	189	27.6	-	-
Diabetes mellitus	116	16.9	-	-
Hypertension	62	9.0	-	-
Autoimmune conditions	7	1.0	-	-
End-stage renal disease	19	2.6	-	-
Malignancy	7	1.0	-	-
Human immunodeficiency virus (*N* = 685)	56	8.2	-	-
No comorbidities	497	72.4	-	-
**Antibiotic exposure (*N* = 405)**	-	-	-	-
Penicillin	116	28.6	-	-
Cephalosporin	143	35.3	-	-
BLBLI	59	14.6	-	-
Carbapenem	50	12.3	-	-
Macrolide	38	9.4	-	-
Other	33	8.1	-	-
One antibiotic class	241	59.5	-	-
One or more antibiotic class	164	40.5	-	-
None	180	26.0	-	-

BLBLI, Beta-lactam and beta-lactamase inhibitor; IQR, interquartile range.

† , Unless otherwise indicated.

The majority of patients were exposed to antibiotics, with ceftriaxone (38%) and amoxicillin-clavulanic acid 241/686 (28.6%) being the most commonly used agents. Notably, carbapenems were prescribed in only 12.3% of the cases.

### Carbapenem-resistant Enterobacterales colonisation

Overall, 85 of 686 patients had a CRE cultured from a rectal swab on either admission or discharge, resulting in an overall CRE prevalence of 12.4% (95% CI: 10.1–15.1). Of these, 46/624 patients had a positive CRE rectal swab at admission (7.4%, 95% CI: 5.6–9.7), and 50/415 patients had a positive CRE rectal swab on discharge (12.0%, 95% CI: 9.1–15.6). Of those patients who tested negative on admission and who had a discharge swab performed, 32/334 patients were CRE positive on discharge (incidence of CRE colonisation of 9.6%). Of those patients who tested positive on admission and had a discharge swab done, 8/19 (42.1%) patients subsequently tested CRE negative on discharge (Table 2).

**TABLE 2:** Rectal swab screening results at admission and discharge.

Rectal swab	Swab positive at admission	Swab negative at admission	Swab not found or rejected at admission	Total
Swab positive at discharge	11	32	7	50
Swab negative at discharge	8	302	55	365
Swab not found or rejected at discharge	27	244	0	271

**Total**	**46**	**578**	**62**	**686**

Note: Total swabs on admission: 624 (46+578). Total swabs on discharge: 415 (50+365). Swabs not found or rejected: 333 (62+271).

There were 87 CREs cultured from 85 rectal swabs. Two swabs yielded two different species of CRE each. *Klebsiella pneumoniae* 69/85 (81.2%) was the most common organism identified (Table 3). Carbapenemases were identified in 55/64 (85.9%) organisms. The most prevalent carbapenemases were OXA-48-like enzymes, detected in 52/64 (81.3%) isolates. New Delhi metallo-β-lactamase was the only other enzyme identified, either in combination with OXA-48 6/64 (9.4%) or as the sole carbapenemase in 3/64 (4.7%) isolates.

**TABLE 3:** Carbapenem-resistant Enterobacterales positive rectal swab results (*N* = 85).

Rectal swab	*n*	%
**CRE organisms** †		
Positive CRE organism cultured on rectal swab		
*Klebsiella pneumoniae*	69	81.2
*Enterobacter cloacae*	9	10.6
*Enterobacter aerogenes*	4	4.7
Other Enterobacter species	2	2.4
*Escherichia coli*	2	2.4
*Citrobacter freundii*	1	1.2
**CPEs results (*n* = 64)** ‡		
OXA-48 only	46	71.9
NDM only	3	4.7
OXA-48 plus NDM	6	9.3
CPE negative	9	14.1

CRE, carbapenem-resistant Enterobacterales; CPE, carbapenemase-producing Enterobacterales; NDM, New Delhi metallo-β-lactamase.

† , Two samples cultured two organisms: *Klebsiella pneumonia* and *Enterobacter cloacae* and *Klebsiella pneumonia* and *Escherichia coli*, respectively;

‡ , Twenty-one swab isolates were not tested for carbapenemase production.

### Risk factors for carbapenem-resistant Enterobacterales colonisation

Of those patients who developed CRE colonisation during their high-care admission, risk factors on univariate analysis included antibiotic exposure (OR: 2.21; 95% CI: 0.98–4.96, *P* = 0.049) and the presence of a CVC (OR: 6.33; 95% CI: 1.78–22.46, *P* = 0.001) (Table 4). Antibiotic exposure was also associated with the acquisition of CRE colonisation in the multivariate analysis (OR: 2.24; 95% CI: 1.12–4.49, *p* = 0.023), but central venous catheterisation was not included in the multivariate model because of data sparsity. No other variables were significantly associated with CRE colonisation in the multivariate logistic regression model.

**TABLE 4:** Risk factors associated with carbapenem-resistant Enterobacterales colonisation and subsequent carbapenem-resistant Enterobacterales infection.

Characteristic	CRE colonisation *n*/*N*	%	Univariate			Multivariate		
			OR	95% CI	*p*	OR	95% CI	*p*
**Risk factors associated with CRE colonisation (*N* = 333)**								
**Age in years**								
< 50	19/214	8.9	1.00	-	-	1.00	-	-
≥ 50	13/119	10.9	1.26	0.60–2.65	0.544	1.17	0.63–2.16	0.627
**Gender**								
Female	14/169	8.3	1.00	-	-	1.00	-	-
Male	18/165	10.9	1.36	0.65–2.83	0.415	1.26	0.69–2.30	0.461
**One or more comorbidities**								
No	28/271	10.3	1.00	-	-	1.00	-	-
Yes	4/63	6.4	0.59	0.20–1.75	0.333	0.49	0.20–1.21	0.121
**Use of device**								
No	9/130	6.9	1.00	-	-	1.00	-	-
Yes	23/204	11.3	1.71	0.76–3.83	0.188	1.26	0.64–2.49	0.503
Central venous catheter	22/139	15.8	6.33	1.78–22.46	0.001	-	-	-
Transurethral catheter	7/96	7.3	0.67	0.28–1.61	0.367	-	-	-
Nasogastric tube	1/13	7.7	0.78	0.10–6.22	0.813	-	-	-
**Received 1 or 2 different antibiotic classes**								
No	9/149	6.0	1.00	-	-	1.00	-	-
Yes	23/185	12.4	2.21	0.98–4.96	0.049	2.24	1.12–4.49	0.023
**Risk factors associated with subsequent CRE infection (*N* = 85)**								
**Age in years**								
< 50 years	9/48	18.8	1.00	-	-	-	-	-
≥ 50 years	5/37	13.5	0.68	0.21–2.22	0.520	-	-	-
**Gender**								
Female	7/43	16.3	1.00	-	-	-	-	-
Male	7/42	16.7	1.03	0.33–3.24	0.962	-	-	-
**One or more comorbidities**								
No	11/59	18.6	1.00	-	-	-	-	-
Yes	3/26	11.5	0.57	0.14–2.24	0.420	-	-	-
**Use of device**								
No	2/18	11.1	1.00	-	-	-	-	-
Yes	12/67	17.9	1.75	0.35–8.62	0.494	-	-	-
**Received 1 or 2 different antibiotic classes**								
No	2/19	10.5	1.00	-	-	-	-	-
Yes	12/66	18.2	1.89	0.38–9.29	0.248	-	-	-

CRE, carbapenem-resistant Enterobacterales; OR, odds ratio; CI, confidence interval.

### Subsequent infections following colonisation

Of the 85 patients who were colonised with a CRE, 18 patients developed a culture-positive infection in the subsequent 180 days (21.2%, 95% CI: 13.1–31.4). These infections occurred at a median of 6.5 days following colonisation (IQR: 3.5–11.5 days), with all but two of these infections occurring within 30 days. Carbapenem-resistant Enterobacterales infections accounted for 14/18 cases (77.8%), giving an odds ratio for CRE infection of 4.0 compared to non-CRE infection (95% CI: 1.3–12.7). Of the CRE infections, urinary tract infections (*n* = 7), blood stream infections (*n* = 5) were the most common sites implicated. One patient with both a positive blood and urine culture yielded identical CPE and cultured organism. Among the subsequent CRE infections, the organism and carbapenemase type were identical to that initially identified by rectal swab. There were no significant differences in risk factors seen between patients who subsequently had a positive CRE culture compared with those who did not (Table 4).

## Discussion

Our study found that 12.4% of patients admitted to a medical high-care ward were colonised with a CRE, with the most frequent organism being *K. pneumoniae* and the most frequently detected carbapenemase being OXA-48-like enzymes. Our study’s CRE prevalence rate aligns with the pooled 16.2% estimate for hospitalised patients from a recent systematic review.^23^ This review found CRE colonisation prevalence to vary considerably by region although, with the prevalence in Asia being highest at approximately 28.4% and the lowest prevalence being seen in Europe and Africa, at 2% – 4%. Carbapenem-resistant Enterobacterales infection rates have increased substantially over the past decade. A point prevalence study in general medical wards in Tygerberg Hospital in 2016 found just a single instance of CRE colonisation from 439 patient samples.^17^ A colonisation prevalence of 5.12% was previously reported from private laboratories in South Africa in 2013 and a recent report indicated that approximately 33% of CRE infections had a previous CRE infection or prior CRE colonisation.^18^ Of note, our calculated CRE colonisation prevalence is likely an underestimate of the true value, as there was a high proportion of missing data, particularly for swabs that were meant to be performed at discharge.

Carbapenem-resistant Enterobacterales colonisation was associated with the presence of a CVC (on univariate analysis) and antibiotic exposure (on both univariate and multivariate analyses). Previous antibiotic exposure has consistently been identified as a risk factor for CRE carriage in the literature, both because antibiotic exposure may select resistant organisms and because eradication of normal gut flora with broad-spectrum antibiotics predisposes to gastrointestinal CRE colonisation.^23^ In keeping with our study, a recent systematic review and meta-analysis found that antibiotic exposure and the use of medical devices such as intravascular catheters were the factors most strongly associated with CRE infections.^24^ Interestingly, the presence of medical comorbidities had no significant association with CRE colonisation in our cohort. This included HIV, which has previously been associated with an increased risk of resistance among Enterobacterales.^25,26^ However, only 8% of our cohort were HIV positive, so it is likely that our study was underpowered to detect this association.

A CPE was identified in most (86%) of the CRE-positive samples, and in the majority of cases, this was OXA-48. Of note, in 10% of the cases where a CPE was detected, this was a combination of OXA-48-like enzyme and an NDM. This combination of CPEs poses significant therapeutic difficulties, as available agents such as colistin have relatively poor efficacy and significant toxicity and novel agents such as aztreonam-avibactam are not available in South Africa currently.

In our cohort, 16.5% of patients who were colonised with CRE subsequently developed a CRE infection within the following 180 days. This was the same percentage identified in a 2015 systematic review of infections following colonisation with CRE, although there was considerable heterogeneity in the individual studies included and the duration of follow-up, with incidences ranging from 0% to 89%.^14^ However, this systematic review only included relevant articles from Europe and North America. To the best of our knowledge, our study is the first to establish a figure for incident CRE infection rate following CRE colonisation anywhere in the African continent.

Of note, CRE colonisation strongly predicted that any subsequent CRE infections within the next 180 days would be because of the same bacterial species and CPE subtype. This finding has clinical implications for clinicians’ choice of empiric antibiotics. The period from the detection of CRE colonisation to the detection of infection was relatively short, with a median of 6.5 days and the majority occurring under 30 days. This may have been an underestimate because CRE colonisation for those testing positive on admission may have occurred before admission to high-care. However, this time course has also been demonstrated in publications from high-income settings, with CRE infection typically occurring between 20 and 90 days post-colonisation.^15,26,27,28^ As was the case in our cohort, these studies have generally not been able to identify specific risk factors associated with the development of CRE infections in patients colonised with CRE.

This study had several limitations. We did not capture data on antibiotic usage and medical devices from study participants outside of their admission to the medical high-care unit nor on recent hospitalisation and transfers from other facilities. Furthermore, there was a high proportion of missing data from the CRE swabs that were meant to be performed on discharge, either because these swabs were not carried out or because the patients died during their admission. These factors may have reduced the strength of the association between these variables and CRE colonisation and/or infection. The morbidity and mortality of CRE infections that occurred following colonisation were also not investigated in this cohort, and thus, the clinical impact of these infections could not be ascertained. The identification of CPEs became temporarily unavailable during the period of 2019; therefore, the number of CPEs identified may have been slightly underrepresented, although not their proportions. The period between colonisation and subsequent CRE infection could only be determined from the date of colonisation ascertainment, and the date of the initial colonisation may have predated admission to the high-care unit. Furthermore, the proportion of CRE-colonised patients who developed a subsequent infection understates the true incidence risk, as the infections had to be associated with a positive culture to be counted. Conversely, despite limiting our definition of subsequent urinary tract infections to those with significant pyuria and pneumonia to those with a high Bartlett score, it was not possible to fully distinguish colonisation from infection. Lastly, this study included data from a single high-care unit, and thus, the generalisability to other hospital contexts in the Southern African region is unclear.

## Conclusion

Our study, conducted in a tertiary-level medical high-care unit, revealed a higher prevalence of CRE colonisation than previously reported in South Africa, as well as a significant incidence of subsequent CRE-driven infections, mostly within 30 days of colonisation. Given the strong link between colonisation and subsequent infection, and the high mortality associated with CRE infections, urgent interventions are required to reduce CRE colonisation rates. In addition, knowledge of CRE rectal colonisation results may assist clinicians in appropriate empiric antibiotic choices if patients present with an infection within 30 days of CRE colonisation.
